# Optimization of the Chitosan-Assisted Extraction for Phillyrin and Forsythoside A from *Forsythia suspensa* Leaves Using Response Surface Methodology

**DOI:** 10.3390/molecules30173528

**Published:** 2025-08-29

**Authors:** Teng Wang, Zexi Zhang, Jiayu Wang, Yuanyuan Fu, Xiaolin Zou, Wei Li, Zhaolun Zhang, Youting Liu, Zhaojun Jia, Zhenguo Wen, Yong Chen

**Affiliations:** 1 Department of Pharmaceutics, Beijing Institute of Petrochemical Technology, Beijing 102627, China; 2 Beijing Uproven Medical Technology Co., Ltd., Beijing 102600, China; 3 Beijing Uproven Institute of Dermatology, Beijing 102600, China

**Keywords:** *Forsythia suspensa* leaves, chitosan, response surface methodology

## Abstract

In this study, a green and efficient extraction methodology was developed by leveraging the unique properties of chitosan—namely its non-toxicity, biocompatibility, and adhesive nature—to enhance the recovery of bioactive ingredients from *Forsythia suspensa* leaves. The core mechanism involves the formation of complexes between chitosan and the target bioactive ingredients, which significantly boosts their extraction efficiency. To substantiate this mechanism, comprehensive characterization was performed using Powder X-ray Diffraction (PXRD), Fourier Transform Infrared Spectroscopy (FT-IR), Differential Scanning Calorimetry (DSC), Scanning Electron Microscopy (SEM), and molecular docking analyses. The results provided robust evidence of a strong interaction between chitosan and the bioactive ingredients, leading to a marked enhancement in both the stability and aqueous solubility of the target compounds. For process optimization, a multi-objective approach was implemented using the Non-dominated Sorting Genetic Algorithm II (NSGA-II) to simultaneously maximize the extraction yields of phillyrin and forsythoside A. The algorithm identified the optimal parameters as a leaf-to-chitosan mass ratio of 10:11.75, a solid-to-liquid ratio of 1:52 g/mL, a temperature of 80 °C, and a duration of 120 min. Under these optimized conditions, the corresponding extraction yields for phillyrin and forsythoside A were 1.68 ± 0.16% and 3.23 ± 0.27%, respectively. These findings collectively indicate that chitosan-assisted extraction represents a highly promising and advanced technology for the sustainable and effective extraction of bioactive ingredients from botanical sources.

## 1. Introduction

**Figure 1:**
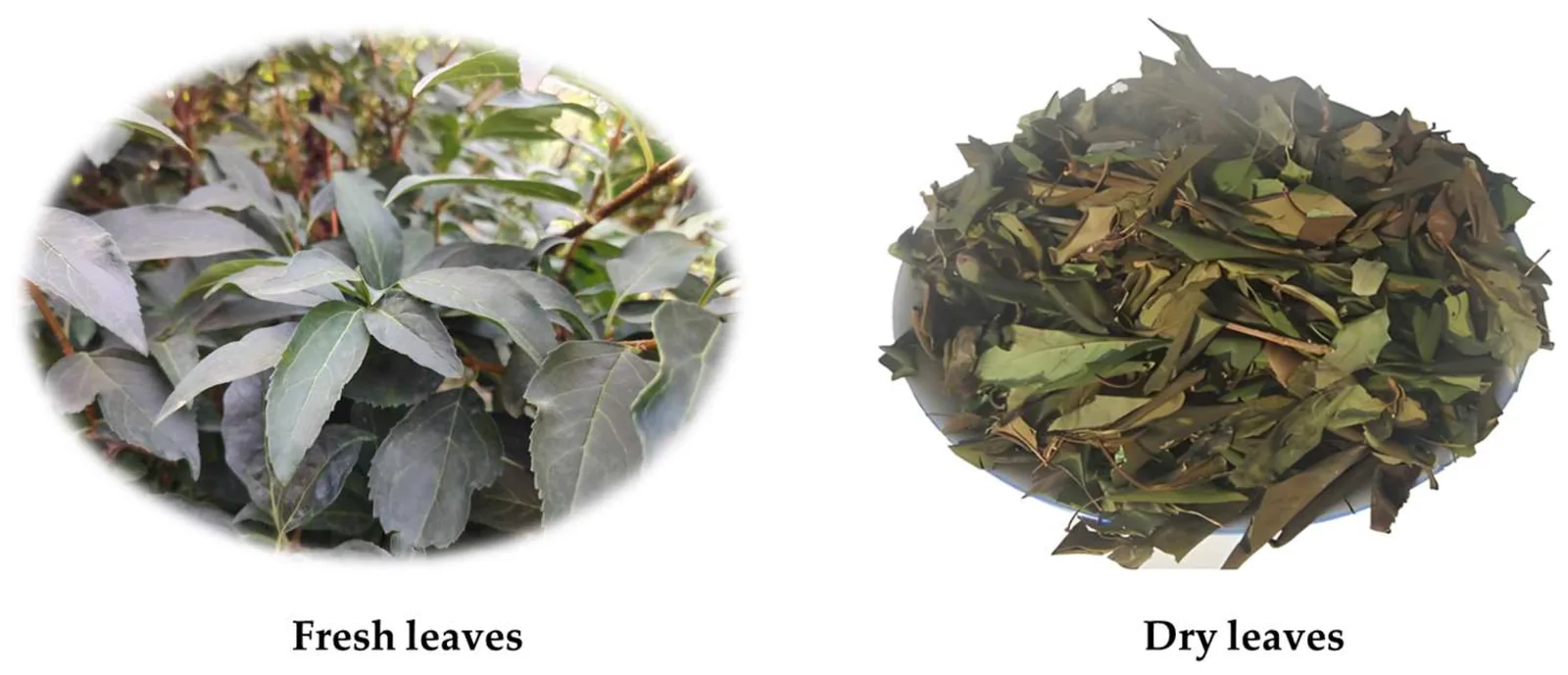
The fresh and dry leaves of *Forsythia suspensa* (Thunb.).

*Forsythia suspensa* (Thunb.) Vahl belongs to the Oleaceae [1,2], and it is extensively cultivated in the East Asian region, encompassing China, South Korea, and Japan, as well as in numerous European nations, as shown in Figure 1. It has been traditionally utilized in Chinese herbal medicine for its efficacy in alleviating pyretic conditions, anti-inflammatory effects, and ability to address specific infectious diseases such as gonorrhea, boils (carbuncles), and erysipelas [3,4,5]. The medicinal properties of *Forsythia suspensa* are attributed to its bioactive ingredients, including phillyrin and forsythoside A, etc., and the bioactive ingredients were confirmed to be low or non-toxic by acute and subchronic tests [6,7]. Hence, these bioactive ingredients have garnered attention for their potential applications in the development of functional foods, cosmeceuticals, nutraceuticals, and pharmaceutical formulations [8,9,10].

The extraction methods for phillyrin and forsythoside A have been reported in several articles, such as the traditional extraction method using organic solvents and hot water [11,12], microwave-assisted extraction [13], ultrasonic-assisted extraction [14], ionic liquids (ILs) [15]. In traditional extraction methods, aqueous solutions often suffer from low extraction efficiency. While organic solvent extraction and ionic liquid extraction methods have improved extraction yields, their toxicity, environmental pollution, and solvent residue make them unsuitable for food and pharmaceutical production. Additionally, novel extraction techniques such as microwave-assisted extraction and ultrasonic-assisted extraction, despite their advantages, are not feasible for large-scale production due to their high costs and complex equipment requirements. Meanwhile, the structures of phillyrin and forsythoside A contain ester bonds and phenolic hydroxyl groups, which are prone to hydrolysis and oxidation under high temperatures and acidic or alkaline conditions, leading to inactivation and consequently poor stability [16]. Therefore, there is an urgent need to develop a simple and feasible method for extracting phillyrin and forsythoside A from *Forsythia suspensa* leaves, enabling their application in products such as foods, cosmetics, and pharmaceuticals.

The development of effective and powerful extraction methods is of pivotal importance for the optimal processing of bioactive ingredients from natural products. Indeed, contemporary techniques are primarily focused on maximizing the extraction yields of specific bioactive ingredients from plant matrices. Furthermore, the use of non-toxic solvents and reagents for this purpose, in the context of a green chemical approach, represents the main objective in natural medicinal chemical research. Chitosan is a linear polysaccharide composed of β-(1→4)-linked D-glucosamine and N-acetyl-D-glucosamine units, which is derived from chitin, a naturally occurring polysaccharide abundant in the exoskeletons of crustaceans [17]. Chitosan is characterized as a cationic polysaccharide, which can be prepared through the deacetylation of chitin under alkaline conditions. Chitosan exhibits a range of physiological functions, including biocompatibility, biodegradability, non-toxicity, and antimicrobial properties, which have led to its extensive application across various fields such as pharmaceuticals, cosmetics, and biomedical sciences [18]. Furthermore, its inherent mucoadhesive properties have enabled its widespread use in the design of mucoadhesive dosage forms, serving as an effective carrier for drug delivery [19]. In aqueous solutions, chitosan exhibits a gel-like state, demonstrating notable adsorption capacity and solution stability. These characteristics also render it useful as an auxiliary extraction agent for the isolation and purification of natural compounds and extracts. Recent years, chitosan-assisted extraction has been used to extract active ingredients from natural products as a green and effective method. For example, Xing et al. [20] examined the influence of chitosan on the extraction of fucoidan from Laminaria japonica. The results demonstrate that chitosan outperforms other auxiliary extractants as the optimal choice, leading to a significant enhancement in the extraction yield of fucoidan.

Chitosan-assisted extraction leverages the ability of chitosan to establish strong intermolecular interactions with the bioactive ingredients of natural products within an aqueous medium. Functioning as a carrier, chitosan facilitates the stable solvation of these bioactive ingredients and significantly enhances their extraction yield [21]. Several critical parameters govern the efficiency of this process, including the solvent pH, extraction temperature, extraction time, solid-to-liquid ratio, and the quantity of chitosan added. Optimization of chitosan-assisted extraction protocols necessitates careful consideration and control of these influential factors [22]. To date, there has been no report about the chitosan-assisted extraction of bioactive ingredients from *Forsythia suspensa* leaves. This study aims to explore this extraction methodology for isolating phillyrin and forsythoside A, with the objective of achieving preparations characterized by high extraction efficiency, enhanced stability, and superior water solubility. The study employed a systematic approach, utilizing single-factor experiments followed by response surface methodology (RSM) to optimize the extraction parameters. Furthermore, the nature of the interactions between chitosan and the isolated bioactive ingredients was elucidated through comprehensive physicochemical characterization employing X-ray Diffraction (XRD), Fourier Transform Infrared Spectroscopy (FTIR), Differential Scanning Calorimetry (DSC), and Scanning Electron Microscopy (SEM).

## 2. Results and Discussion

### 2.1. Analysis of the Results of the Single-Factor Experiments

#### 2.1.1. Effect of the Extraction Time on the Yields of Phillyrin and Forsythoside A

**Figure 2:**
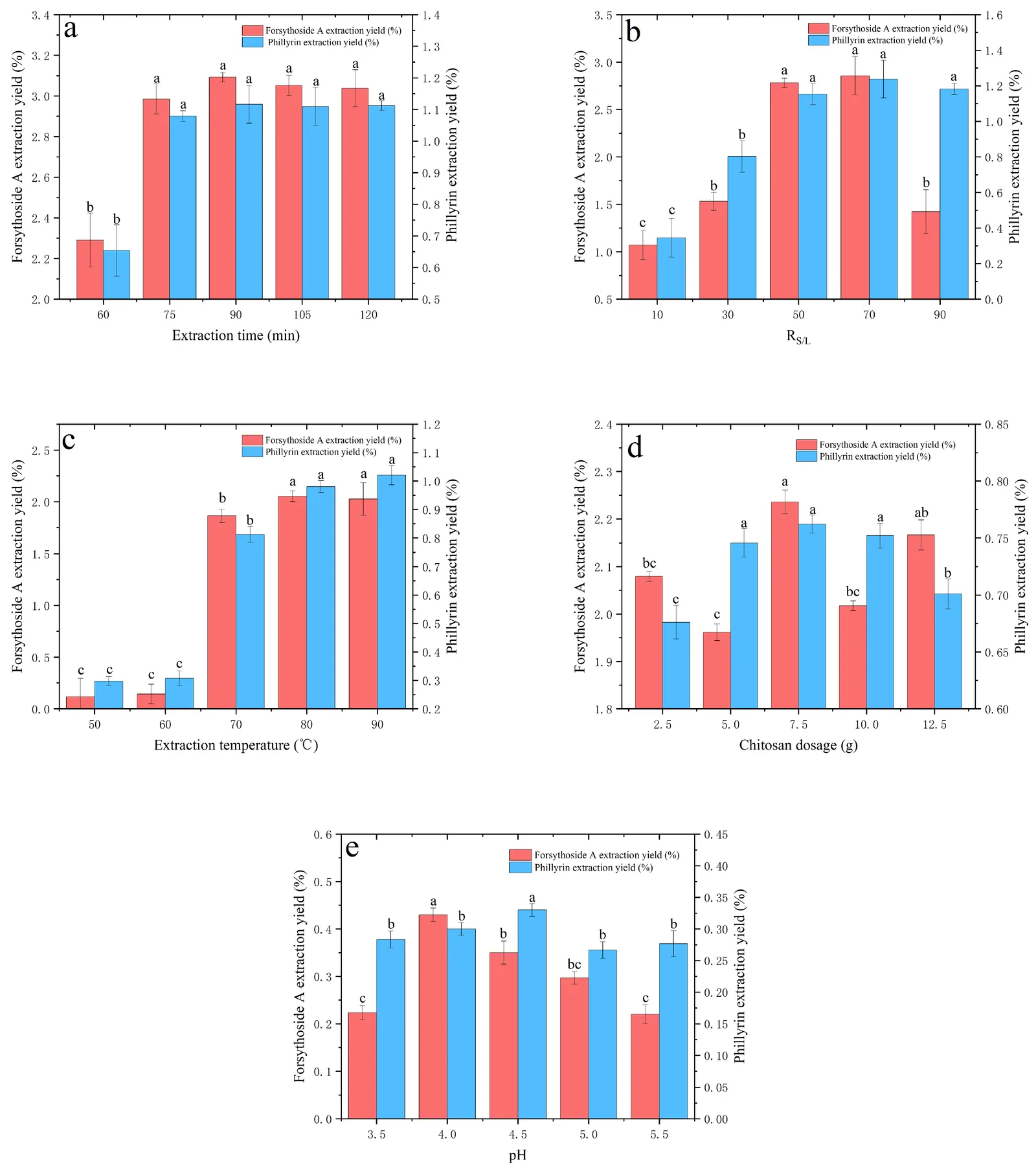
Chitosan-assisted extraction single-factor influence diagram. (**a**) Extraction time; (**b**) solid–liquid ratio (R_S/L_); (**c**) extraction temperature; (**d**) chitosan dosage; and (**e**) extraction pH. Data are expressed as the mean ± SD of *n* = 3 samples. Different lowercase letters (a, b, and c) in the same figure indicate statistically significant differences (*p* < 0.05).

Figure 2 illustrates the influence of extraction time on the yields of bioactive ingredients from *Forsythia suspensa* leaves. As depicted in Figure 2a, the yields of both phillyrin and forsythoside A exhibited a marked increase within the 60 to 90 min interval, reaching peak values approximately at the 90 min mark before subsequently plateauing. Notably, the yield of forsythoside A displayed a slight decline post 90 min. This phenomenon is tentatively attributed to the potential thermal hydrolysis of forsythoside A under prolonged elevated extraction temperatures [1]. Considering that the maximal extraction yields for both compounds were achieved around the 90 min point, an extraction time of 90 min was judiciously selected as the central point for the response surface methodology (RSM) optimization, with a broader range of 60 to 120 min being explored for further investigation.

#### 2.1.2. Effect of Solid–Liquid Ratio (R_S/L_) on the Yields of Phillyrin and Forsythoside A

The impact of the solid-to-liquid ratio (R_S/L_) on the yields of bioactive ingredients from *Forsythia suspensa* leaves is presented in Figure 2b. As observed, the extraction yield of phillyrin demonstrated a significant increase as the R_S/L_ ratio was elevated from 1:10 to 1:70, reaching a maximum at R_S/L_ 1:70. This may be attributed to the higher liquid-to-material ratio, which creates a larger concentration gradient between the interior of the plant cells and the external solvent, thereby facilitating the rapid diffusion of bioactive ingredients [23]. Beyond this point, further increases in the ratio resulted in only marginal variations in extraction efficiency. In contrast, the yield of forsythoside A exhibited a rapid escalation within the same R_S/L_ range (1:10 to 1:70) but subsequently declined sharply thereafter. This decline is hypothesized to correlate with the fixed amount of chitosan employed; specifically, the increasing solvent volume, which dilutes the chitosan concentration, likely reduced its binding efficacy towards forsythoside A, consequently lowering the extraction efficiency. Given that the yield for phillyrin peaked most distinctly at R_S/L_ 1:50, and considering the overall trends for both compounds, this ratio was selected as the central point for the response surface methodology (RSM) optimization, with a broader investigation range set between 1:30 and 1:90.

#### 2.1.3. Effect of the Extraction Temperature on the Yields of Phillyrin and Forsythoside A

Figure 2c illustrates the influence of the extraction temperature on the yields of the bioactive ingredients from *Forsythia suspensa* leaves. As evident from Figure 2c, the yields of both phillyrin and forsythoside A exhibited a marked increase within the temperature range of 60 to 80 °C, indicating that temperature is a significant factor affecting the extraction efficiency. Beyond 80 °C, the extraction yield of phillyrin continued to increase with rising temperature, whereas that of forsythoside A exhibited a decline. The observed increase between 60 and 80 °C is likely attributable to the enhanced molecular mobility of the active ingredients within the leaf matrix as the temperature rises. This increased mobility likely improves their solubility in the extraction solvent. Concurrently, higher temperatures may also facilitate greater interaction between chitosan and the target compounds within the leaves, thereby enhancing the overall extraction yield. Considering these dynamics and aiming for optimal extraction conditions, 70 °C was ultimately selected as the central temperature point for this study, with a subsequent investigation range established between 70 and 90 °C.

#### 2.1.4. Effect of the Chitosan Dosage on the Yields of Phillyrin and Forsythoside A

The influence of the chitosan dosage on the yields of the bioactive ingredients from *Forsythia suspensa* leaves is depicted in Figure 2d. As observed, the extraction yield of phillyrin increased progressively with the increasing chitosan concentration, reaching a maximum within the range of 2.5 to 7.5 g, subsequently plateauing between 5.0 and 10.0 g, and then declining from 10.0 to 12.5 g. In contrast, the yield of forsythoside A initially decreased as chitosan was added, experienced a rapid increase between 5.0 and 7.5 g to attain its peak, followed by a decline, and then exhibited a resurgence at 10.0 g, indicating a non-monotonic relationship. The data analysis revealed divergent trends in the extraction yields of these two compounds relative to the chitosan dosage. It is speculated that this divergent behavior may stem from the varying binding affinities of chitosan towards phillyrin and forsythoside A, consequently influencing their respective extraction kinetics. Considering these observations, a chitosan dosage of 7.5 g was selected as the central point for the response surface methodology (RSM) optimization, with a subsequent investigation range established between 2.5 and 12.5 g.

#### 2.1.5. Effect of the Solution pH on the Yields of Phillyrin and Forsythoside A

The solvent pH also influences the extraction efficiency mediated by chitosan. Under acidic conditions, chitosan undergoes full protonation, which significantly enhances its aqueous solubility and promotes the exposure of active functional groups, thereby facilitating the extraction of bioactive ingredients. In the present study, given the safety and stability requirements for Forsythia extract applications, glycyrrhizic acid—a naturally derived phytochemical—was strategically selected as the pH-modulating agent for the extraction system. This choice aligns with the principles of green extraction while ensuring biocompatibility.

Figure 2e illustrates the impact of the solution pH on the yields of the bioactive ingredients from *Forsythia suspensa* leaves. As observed, the extraction yield of phillyrin exhibited minimal fluctuation within the pH range of 3.5 to 5.5, with variations remaining below ±0.3%. This suggests that, under these experimental conditions, the solution pH has negligible influence on the extraction of both phillyrin and forsythoside A. This phenomenon can be attributed to the fact that chitosan remains fully protonated across this pH range, while the competing effects of solution viscosity and solute solubility may counterbalance the variations in binding affinity. Consequently, the pH was maintained at 4.0 for the chitosan-assisted extraction process in this study.

### 2.2. Box–Behnken Design (BBD) for Chitosan-Assisted Extraction

#### 2.2.1. Model Building and Statistical Analysis

Based on the single-parameter studies, a Box–Behnken design (BBD) was employed to investigate the extraction process under the following conditions: extraction time (X_1_) ranging from 60 to 120 min, the solid-to-liquid ratio (R_S/L_, X_2_) ranging from 1:30 to 1:90, extraction temperature (X_3_) ranging from 70 to 90 °C, and chitosan dosage (X_4_) ranging from 2.5 to 12.5 g/10 g *Forsythia suspensa* leaves. As shown in Table 1, a total of 29 experimental runs were conducted to optimize the four independent parameters in the current BBD. These parameters were designated as Y_1_ and Y_2_: the response as the yield of phillyrin and forsythoside A, X_1_: extraction time (60–120 min), X_2_: solid-to-liquid ratio (1:30–1:90), X_3_: extraction temperature (70–90 °C), and X_4_: chitosan dosage (2.5–12.5 g).

The experimental data, encompassing both the process parameters and corresponding response values, were analyzed using Design-Expert 8 software. This analysis aimed to develop second-order polynomial regression models for the two responses: the yields of phillyrin (Y_1_) and forsythoside A (Y_2_). These models mathematically describe the relationships between the response variables and the independent process parameters (X_1_, X_2_, X_3_, X_4_), as presented in the following equations:

(1) $$Y_{1}=-5.47544-0.018890X_{1}+0.02215X_{2}+0.14871X_{3}-0.11221X_{4}+5.65947\times 10^{-5}X_{1}X_{2}+\;4.82302\times 10^{-5}X_{1}X_{3}+3.17269\times 10^{-4}X_{1}X_{4}-1.02247\times 10^{-4}X_{2}X_{3}+6.79131\times 10^{-4}X_{2}X_{4}+\;1.10674\times 10^{-3}X_{3}X_{4}+6.28438\times 10^{-5}X_{1}^{2}-1.61458\times 10^{-4}X_{2}^{2}-7.53775\times 10^{-4}X_{3}^{2}-1.74709\times 10^{-3}X_{4}^{2}.$$

(2) $$Y_{2}=-40.42172+9.66931\times 10^{-3}X_{1}+0.1306X_{2}+1.02704X_{3}-0.29562X_{4}+6.43884\times 10^{-4}X_{1}X_{2}+\;2.97787\times 10^{-6}X_{1}X_{3}+3.56370\times 10^{-3}X_{1}X_{4}-1.07298\times 10^{-3}X_{2}X_{3}-1.87530\times 10^{-4}X_{2}X_{4}+\;2.31786\times 10^{-3}X_{3}X_{4}-3.77782\times 10^{-4}X_{1}^{2}-1.14486\times 10^{-3}X_{2}^{2}-6.14174\times 10^{-3}X_{3}^{2}-0.013204X_{4}^{2}.$$

To validate the statistical significance of the established models, analysis of variance (ANOVA) was performed. The significance of each coefficient in the model was assessed using the F-test and corresponding *p*-values, as presented in Table 2 and Table 3. The ANOVA results for the extraction efficiency models of phillyrin and forsythoside A revealed F-values of 43.92 and 41.27, respectively. The associated *p*-values were both less than 0.05, demonstrating that both regression models were statistically significant. Furthermore, the adequate precision values, which assess the signal-to-noise ratio, were calculated to be 24.958 and 19.878 for the respective models. Since these values exceeded the critical threshold of 4, it confirms that the models possess a sufficient signal-to-noise ratio, ensuring their reliability and robustness for further analysis and prediction [24].

The analysis of variance for the response surface model is presented in Table 4 and Table 5. For the response surface model of phillyrin, it is evident that the linear coefficients (X_2_, X_3_), and the quadratic term coefficients (X_3_^2^, X_4_^2^), were statistically significant as shown in Table 4. “Prob > F” values less than 0.001 denote that the model terms are significant.

For the response surface model of forsythoside A, the linear coefficients (X_3_), the quadratic term coefficients (X_1_^2^, X_2_^2^, X_3_^2^, X_4_^2^), and the interaction coefficients (X_1_X_2_, X_1_X_4_) were statistically significant as shown in Table 5. Among these linear coefficients, X_3_, X_2_^2^, X_3_^2^ and X_1_X_4_ were identified as significant model terms with very small *p*-values (*p* < 0.0001). Additionally, X_1_X_2_, X_1_^2^, and X_4_^2^ were determined to be important factors with small *p*-values (*p* < 0.01).

Comparative analysis of the F-values for each factor, as shown in Table 4 and Table 5, further reveals that factor X_3_ demonstrates the most pronounced effect on the yields of phillyrin and forsythoside A within this regression model. This is substantiated by F-values of 521.21 and 452.89, respectively. The significance order of the factors for phillyrin is as follows: extraction temperature (X_3_) > solid-to-liquid ratio (X_2_) > chitosan dosage (X_4_) > extraction time (X_1_), while the significance order of the factors for forsythoside A is extraction temperature (X_3_) > chitosan dosage (X_4_) > solid-to-liquid ratio (X_2_) > extraction time (X_1_) [25].

#### 2.2.2. Optimization of the Procedure

**Figure 3:**
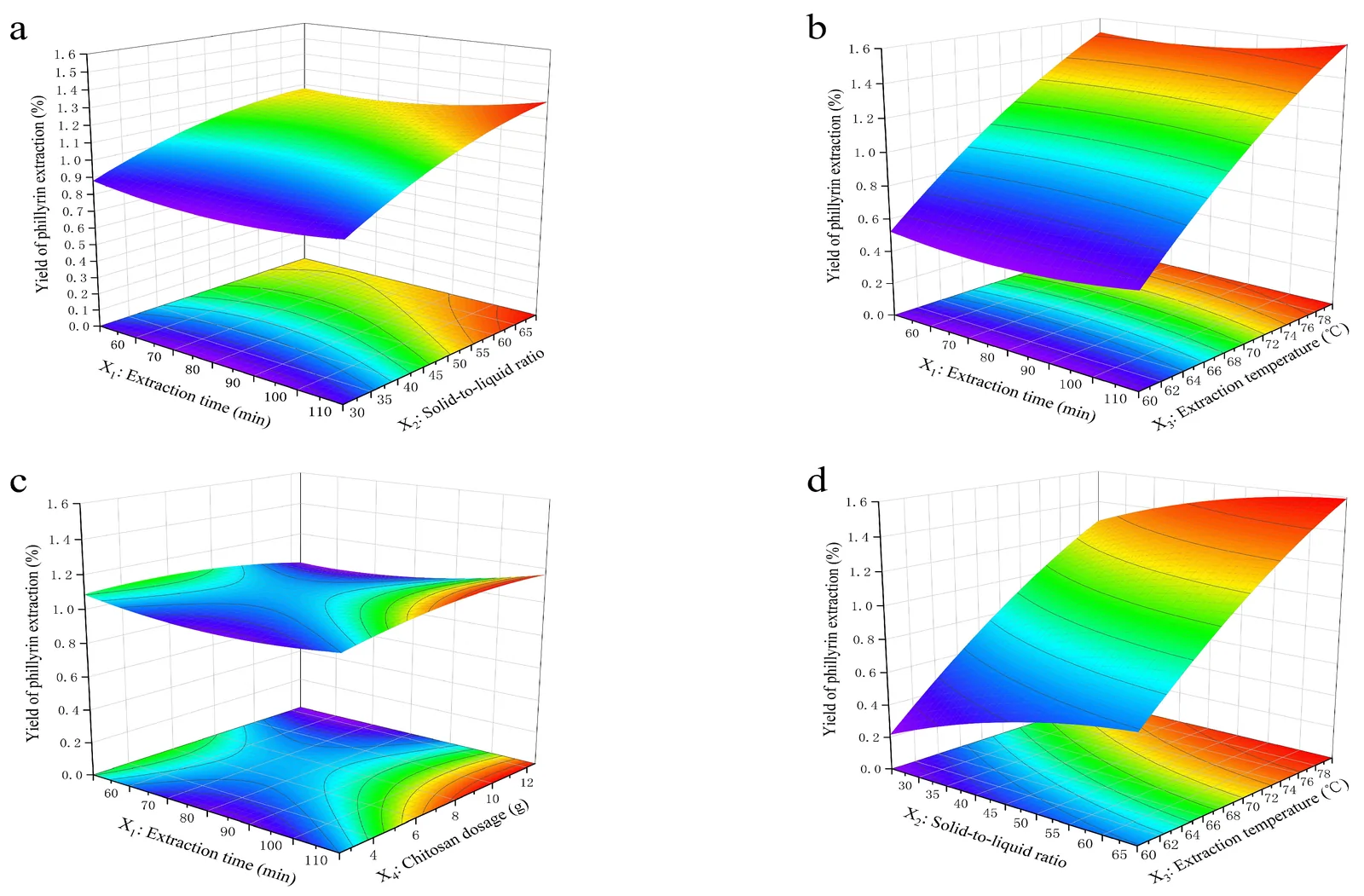

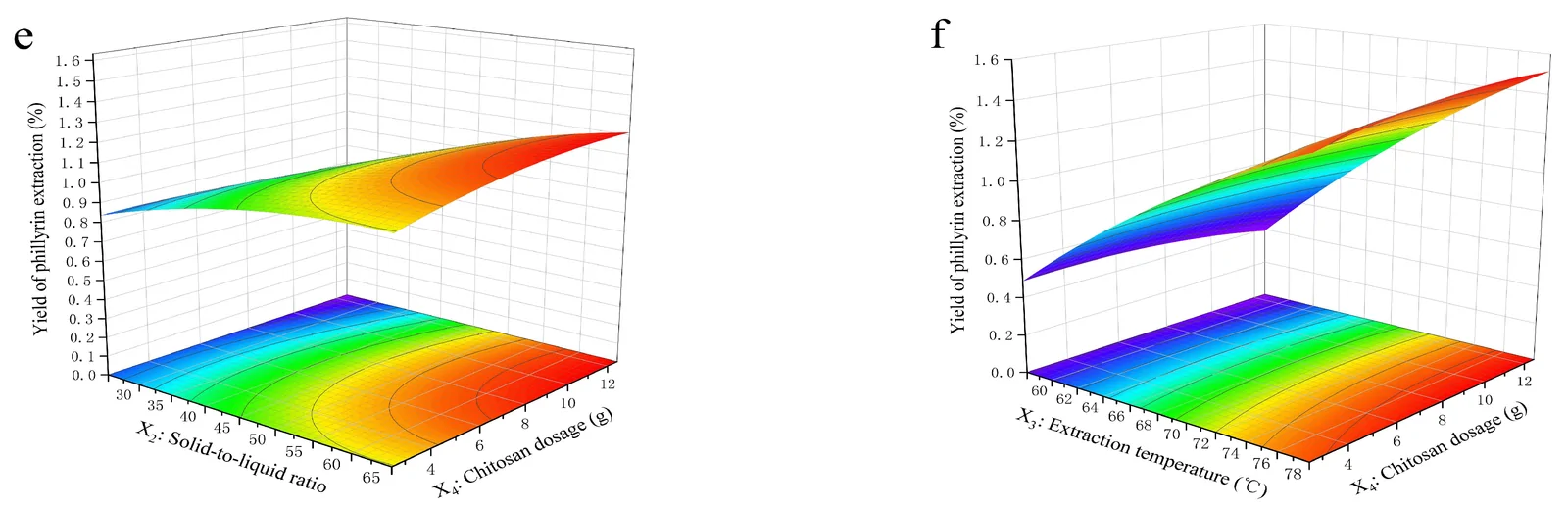
Three-dimensional response plots of the interaction effect of the parameter variables of phillyrin on the extraction efficiency. (**a**) Interaction of the extraction time with R_S/L_; (**b**) interaction of the extraction time with the extraction temperature; (**c**) interaction of the extraction time with the chitosan dosage; (**d**) interaction of R_S/L_ with the extraction temperature; (**e**) interaction of R_S/L_ with the chitosan dosage; and (**f**) interaction of the extraction temperature with the chitosan dosage.

**Figure 4:**
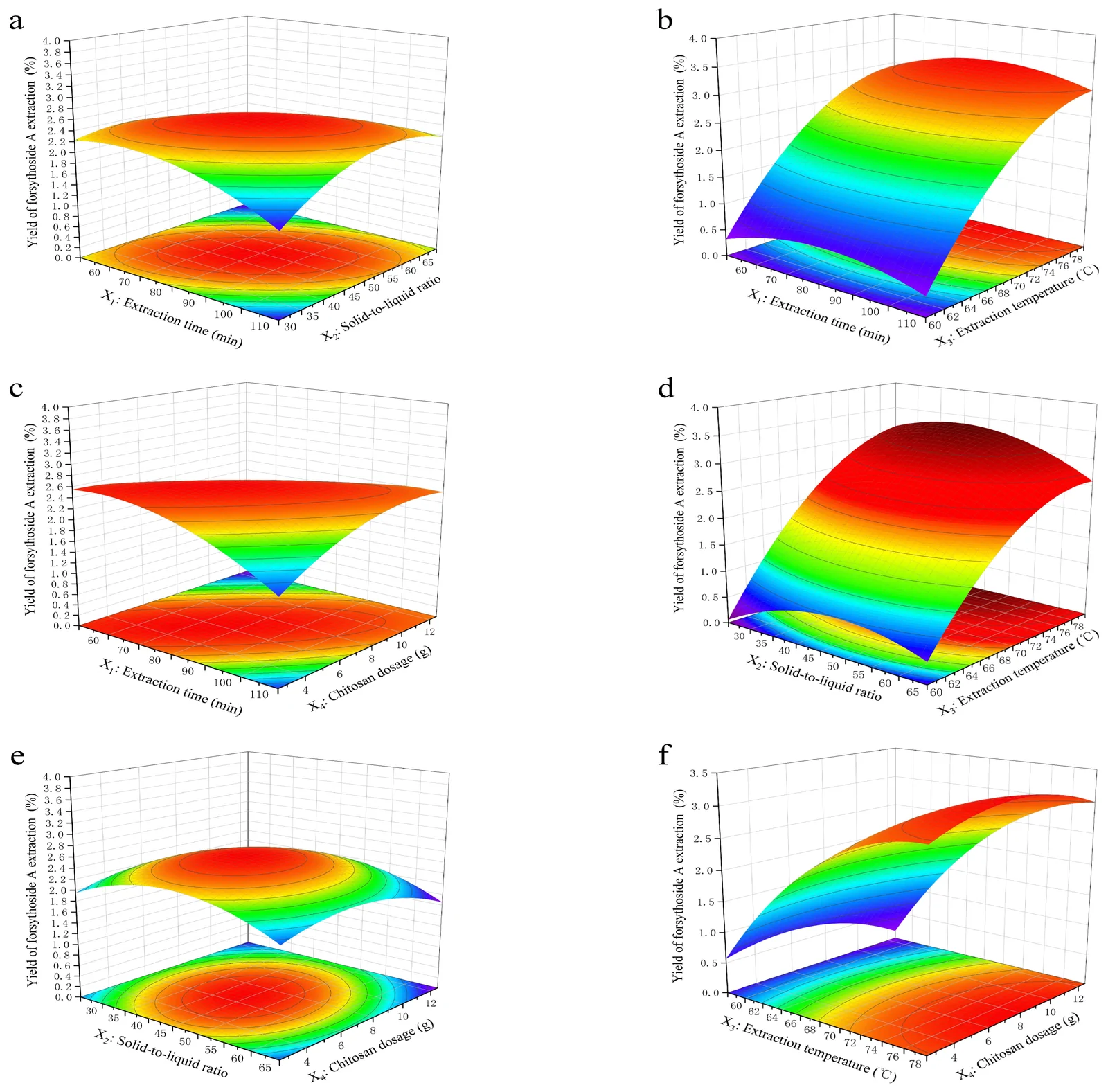
Three-dimensional response plots of the interaction effect of the parameter variables of forsythoside A on the extraction efficiency. (**a**) Interaction of the extraction time with R_S/L_; (**b**) interaction of the extraction time with the extraction temperature; (**c**) interaction of the extraction time with the chitosan dosage; (**d**) interaction of R_S/L_ with the extraction temperature; (**e**) interaction of R_S/L_ with the chitosan dosage; and (**f**) interaction of the extraction temperature with the chitosan dosage.

Figure 3 and Figure 4 present the interaction effects between the variables and the 3D response surface as graphical representations of the regression equation. These visual tools facilitate the interpretation of the relationship between the response variables and the experimental levels of each variable, as well as the nature of the interactions between the two test variables. The contour plot shapes, whether circular or elliptical, indicate the significance of the interactions between variables: circular shapes suggest negligible interactions, whereas elliptical shapes indicate significant interactions [26,27]. In the 3D response surface generated by the model for the yields of phillyrin and forsythoside A, the relationships between two variables are depicted on a 3D surface plot, while the other variables are held at a zero level.

As illustrated in Figure 3, the extraction temperature (X_3_) was the most significant factor influencing the yield of phillyrin. The interactive effects of the parameters on the extraction yield are evident in Figure 2b,d, where the chitosan dosage (X_4_) is held constant at its zero level. Specifically, when both the extraction time (X_1_) and the solid-to-liquid ratio (X_2_) were maintained at lower levels, the yield of phillyrin increased markedly with a rise in the extraction temperature (X_3_).

The influence of various extraction parameters on the yield of forsythoside A mirrored that of phillyrin, with extraction temperature (X_3_) emerging as the most influential variable. The interactive effects, as depicted in Figure 3b,d (with chitosan dosage (X_4_) fixed), reveal a pronounced enhancement in forsythoside A yield corresponding to an increase in the extraction temperature (X_3_), particularly when the extraction time (X_1_) and solid-to-liquid ratio (X_2_) are constrained to their lower levels. The 3D response surface and contour plots illustrated in Figure 4f further substantiate that the yield is profoundly affected by both the extraction temperature (X_3_) and chitosan dosage (X_4_), signifying that the extraction temperature and chitosan dosage are the most crucial factors. This conclusion is in strong agreement with the statistical significance indicated by the model’s F-value.

### 2.3. NSGA-II Algorithm for Bi-Objective Optimization

While the response surface methodology is capable of optimizing the extraction conditions for phillyrin and forsythoside A individually (as shown in Table 6), it cannot identify a set of conditions that achieves a comprehensive optimization for both target compounds simultaneously. In contrast, the Non-dominated Sorting Genetic Algorithm II (NSGA-II), a classical multi-objective optimization algorithm proposed by Deb in 2000, is specifically designed to address such problems [28]. Its core principle is to identify a set of optimal compromise solutions—known as the Pareto optimal set—among multiple conflicting objective functions. The NSGA-II achieves a balance between convergence and diversity in the solution set through three key mechanisms: fast non-dominated sorting, crowding distance comparison, and an elite preservation strategy [29]. Recent advancements have seen the NSGA-II algorithm increasingly employed to optimize methodologies for the co-extraction and separation of various bioactive ingredients from natural product matrices [30,31,32].

The optimal extraction yields of phillyrin and forsythoside A, obtained by inputting Equations (1) and (2) into the NSGA-II algorithm, were 1.69% and 3.23%, as shown in Table 6. Under these optimal conditions, the extraction time, solid-to-liquid ratio (R_S/L_), extraction temperature, and chitosan dosage were determined to be 119.99 min, 1:52.06, 79.99 °C, and 11.75 g, respectively. Meanwhile, under these optimal conditions, the predicted extraction yields for phillyrin and forsythoside A, calculated from Equations (1) and (2), were 1.69% and 3.22%, respectively. These values are in strong agreement with the results optimized by the NSGA-II algorithm.

### 2.4. Validation of the Model

To validate the optimization, a confirmatory experiment was performed under slightly modified conditions: an extraction time of 120 min, a solid-to-liquid ratio of 1:52, an extraction temperature of 80 °C, and the chitosan dosage of 11.75 g. The actual yields of phillyrin and forsythoside A obtained from the experimental set were 1.68 ± 0.16% and 3.23 ± 0.27% (*n* = 3), which was in good agreement with the predicted value of the model equation. This confirmed the adequacy of the NSGA-II algorithm for the optimization process. To further evaluate the effect of the chitosan dosage on the yields of phillyrin and forsythoside A, a confirmatory experiment was conducted under the same optimized conditions but without the addition of chitosan. The results, presented in Table 7, indicate that the inclusion of chitosan significantly enhanced the yields of both phillyrin and forsythoside A.

In 2020, Li et al. [33] provided a review of past research on the extraction, separation, structural identification, and biological activities of bioactive ingredients from *Forsythia suspensa* leaves conducted by various investigators. As summarized in this review, the maximum yields of phillyrin obtained using aqueous and ethanolic solvents were reported by Wang et al. and Liang et al., corresponding to 0.39% and 1.84%, respectively [34,35]. For the extraction of forsythoside A, Fang et al. [13] and Sun et al. [15] reported yields of 2.75% and 10.74%, respectively, utilizing microwave-assisted aqueous alcohol extraction and ionic liquid-based ultrasonic-assisted extraction (ILUAE). Compared with the aforementioned literature, the chitosan-assisted extraction methodology presents a substantial improvement in extraction efficiency within an aqueous medium. A key advantage of this technique is the elimination of organic solvents, such as ethanol and ionic liquids, thereby enhancing the environmental compatibility and safety of the process. Given its non-toxic nature and favorable biocompatibility, this method is particularly well-suited for applications in the manufacturing of food, pharmaceutical, and cosmetic products intended for human use. Moreover, this method avoids the complexities and potential drawbacks inherent in equipment-intensive techniques, such as ultrasound- and microwave-assisted extraction.

### 2.5. Characterization of Chitosan-Assisted Extraction of Natural Products from Forsythia suspensa Leaves

#### 2.5.1. Fourier Transform Infrared Spectroscopy (FT-IR) Analysis

**Figure 5:**
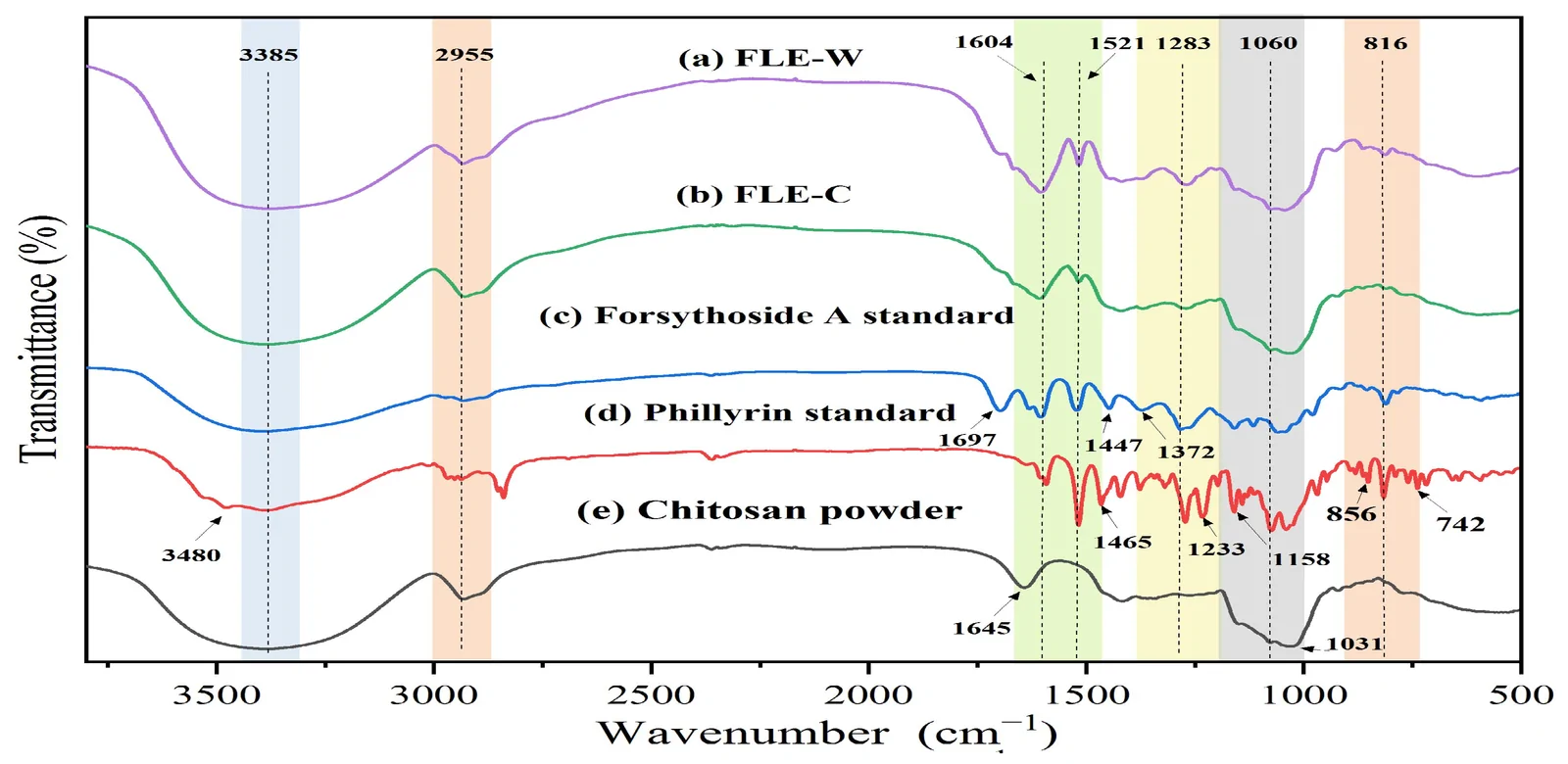
FT-IR spectra of (a) water extract of *Forsythia suspensa* leaves (FLE-W), (b) chitosan-assisted extract of *Forsythia suspensa* leaves (FLE-C), (c) forsythoside A standard, (d) phillyrin standard, and (e) chitosan powder.

FT-IR spectra of the water extract of *Forsythia suspensa* leaves (FLE-W), chitosan-assisted extract of *Forsythia suspensa* leaves (FLE-C), forsythoside A standard, phillyrin standard, and chitosan powder are shown in Figure 5. As shown in the figure, the spectra of the FLE-W, FLE-C, phillyrin standard, and forsythoside A standard collectively displayed key absorption bands indicative of their functional groups. Notably, a broad and intense band centered around 3385 cm^−1^ is attributed to the O-H stretching vibration, while the absorption at 2955 cm^−1^ is assigned to the C-H stretching vibration of aliphatic groups. It also shows high intensity absorption peaks at 1604 cm^−1^ and 1521 cm^−1^, which is the stretching vibration of the benzene ring skeleton. The significant peaks at 1283 cm^−1^ and 1060 cm^−1^ were assigned to the C-O stretching vibration. There was a pair of double substituted benzene ring vibration peaks at 816 cm^−1^. The IR spectrum of chitosan was characterized by the prominent bands at 1660 cm^−1^ corresponding to the amide group stretching vibration. There was a particularly significant characteristic absorption peak at 1060 cm^−1^ that can be assigned to the C-O-C stretching vibration.

As can be seen from the figure, compared to FLE-W, phillyrin, and forsythoside A, the stretching vibration of the benzene ring skeleton of FLE-C is significantly weakened at 1521 cm^−1^, especially at 1465 cm^−1^, 1447 cm^−1^, 1372 cm^−1^, 1233 cm^−1^, and 816 cm^−1^, where the characteristic peaks almost disappear completely in FLE-C. The stretching vibration of the benzene ring skeleton of FLE-C is also significantly weakened at 1283 cm^−1^ and 1060 cm^−1^. Other absorption peaks of FLE-C, for example at 1283 cm^−1^, were also attenuated compared to FLE-W. FT-IR spectra showed that the interaction between host and guest molecules resulted in the attenuation of the characteristic peaks of some active substances [36].

#### 2.5.2. Powder X-Ray Diffraction (XRD) Analysis

XRD studies are usually used to identify the physical state of the molecules and characterize the conjugates. The formation of conjugates between chitosan and crystalline guest molecules can be confirmed by the disappearance of the crystalline state of the guest molecule upon binding to chitosan [36].

**Figure 6:**
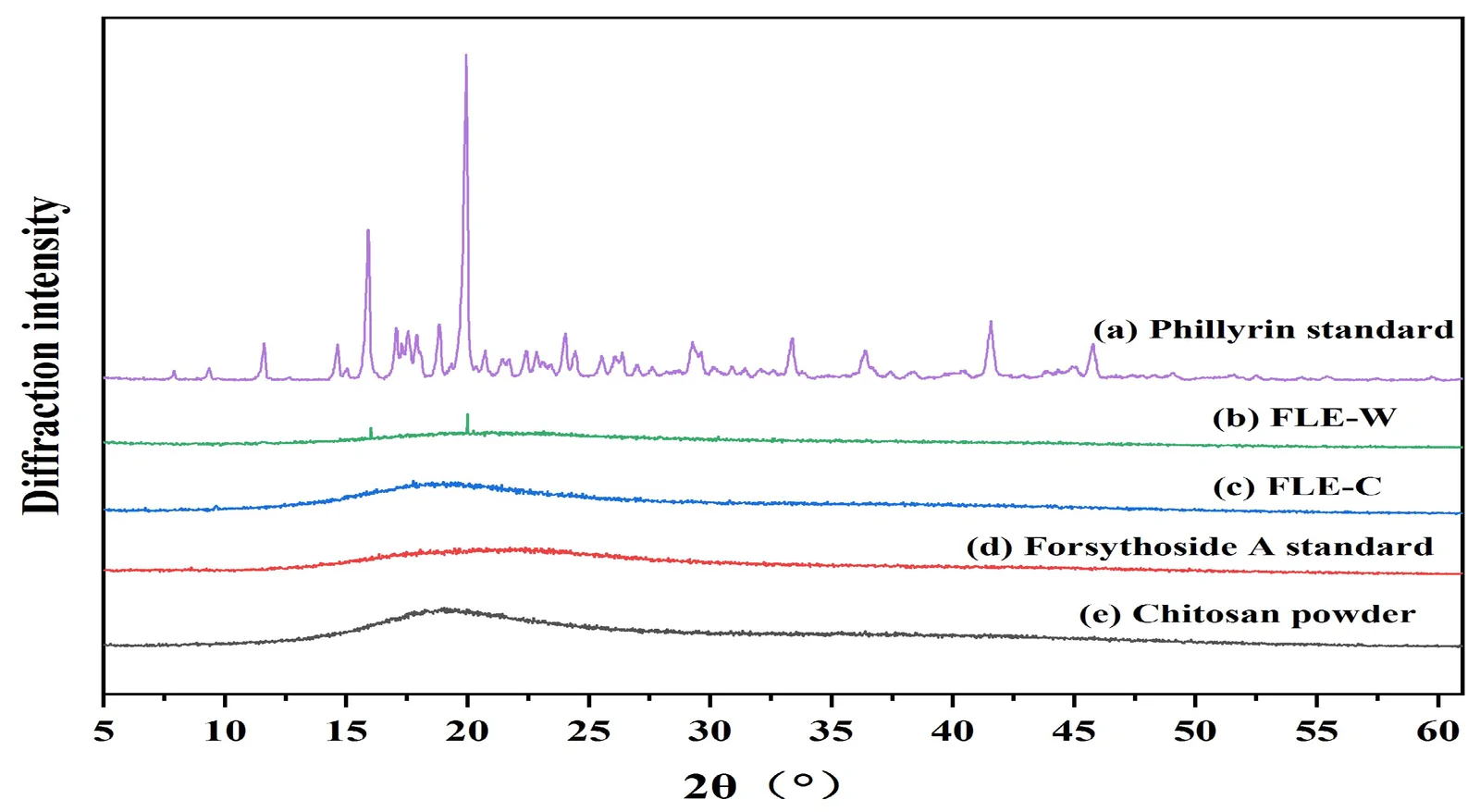
Powder XRD diffractogram of (a) phillyrin standard, (b) water extract of *Forsythia suspensa* leaves (FLE-W), (c) chitosan-assisted extract of *Forsythia suspensa* leaves (FLE-C), (d) forsythoside A standard, and (e) chitosan powder.

As illustrated in Figure 6, phillyrin exhibits a distinct crystalline structure, whereas forsythoside A and chitosan are amorphous. The aqueous extract of *Forsythia suspensa* leaves displays characteristic diffraction peaks at 2θ angles of 16.7° and 20.19°, which can be attributed to the presence of phillyrin. In contrast, the chitosan-assisted extract shows a broad halo peak centered at a 2θ angle of 18.73°, with the absence of any sharp crystalline peaks, indicative of an amorphous structure. This finding is consistent with the results reported in the literature [37]. These observations further confirm that an interaction occurs between chitosan and the bioactive ingredients of *Forsythia suspensa* leaves.

#### 2.5.3. Differential Scanning Calorimetry (DSC) Analysis

**Figure 7:**
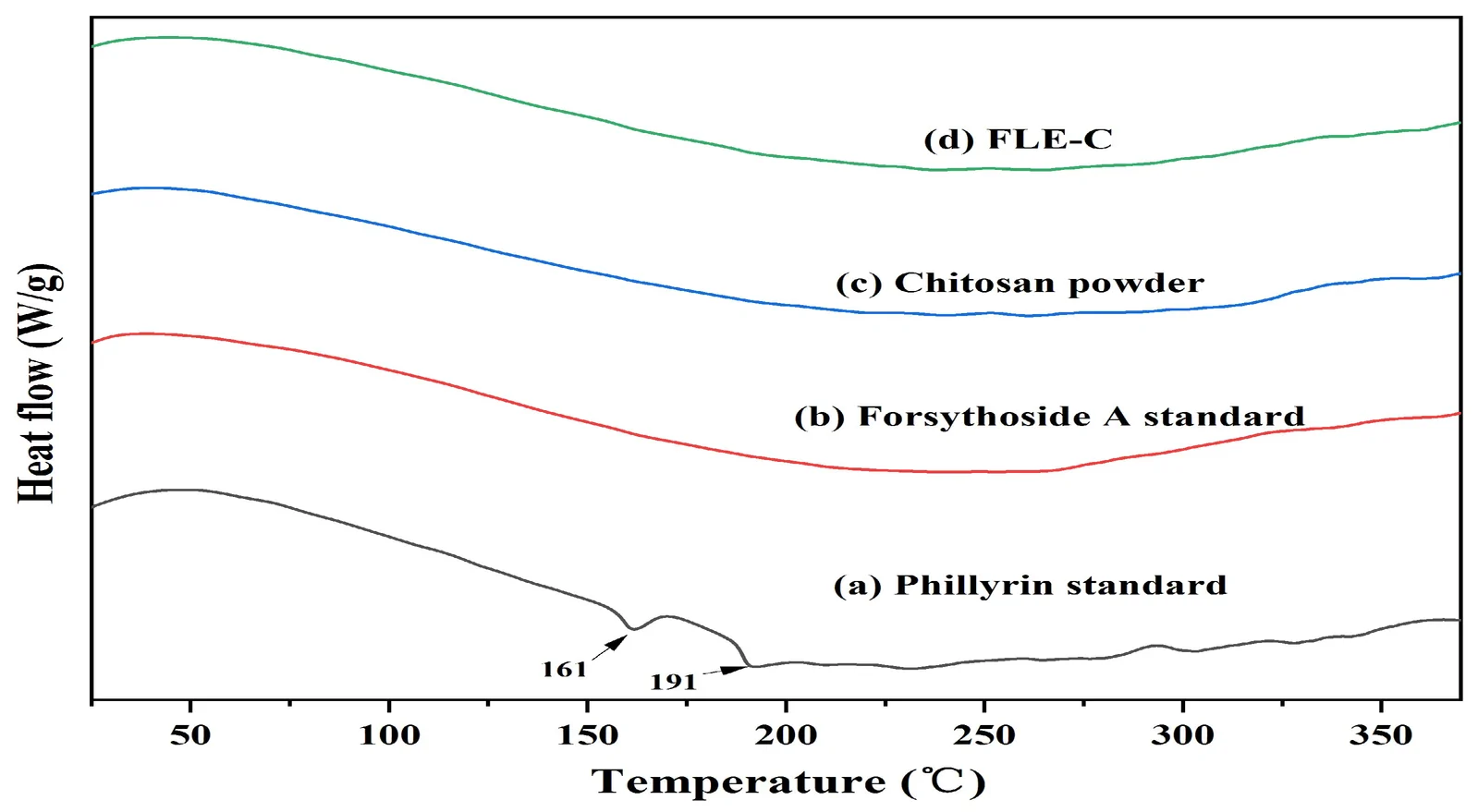
DSC result of (a) phillyrin standard, (b) forsythoside A standard, (c) chitosan powder, and (d) chitosan-assisted extract of *Forsythia suspensa* leaves (FLE-C).

The differential scanning calorimetry curves for the phillyrin standard, forsythoside A standard, chitosan, and the chitosan-assisted extract are presented in Figure 7. The DSC analysis confirmed that phillyrin exhibits a crystalline structure. Correspondingly, its DSC thermogram revealed two distinct endothermic peaks at approximately 161 °C and 191 °C, attributable to a solid–solid phase transition and the melting point, respectively. In contrast, the DSC profiles of both forsythoside A and chitosan were devoid of any discernible thermal events, indicating their amorphous or non-crystalline nature. Notably, in the DSC curve of the chitosan-assisted extract, the characteristic peaks of phillyrin were no longer present; instead, only minor thermal fluctuations were observed. This disappearance strongly suggests an interaction between phillyrin and chitosan, likely leading to the formation of a new complex that masks the individual thermal properties of the crystalline compound. Therefore, these results provide compelling evidence for an interaction between the bioactive components of *Forsythia suspensa* leaves and chitosan during the extraction process.

#### 2.5.4. Scanning Electron Microscopy (SEM) Analysis

**Figure 8:**
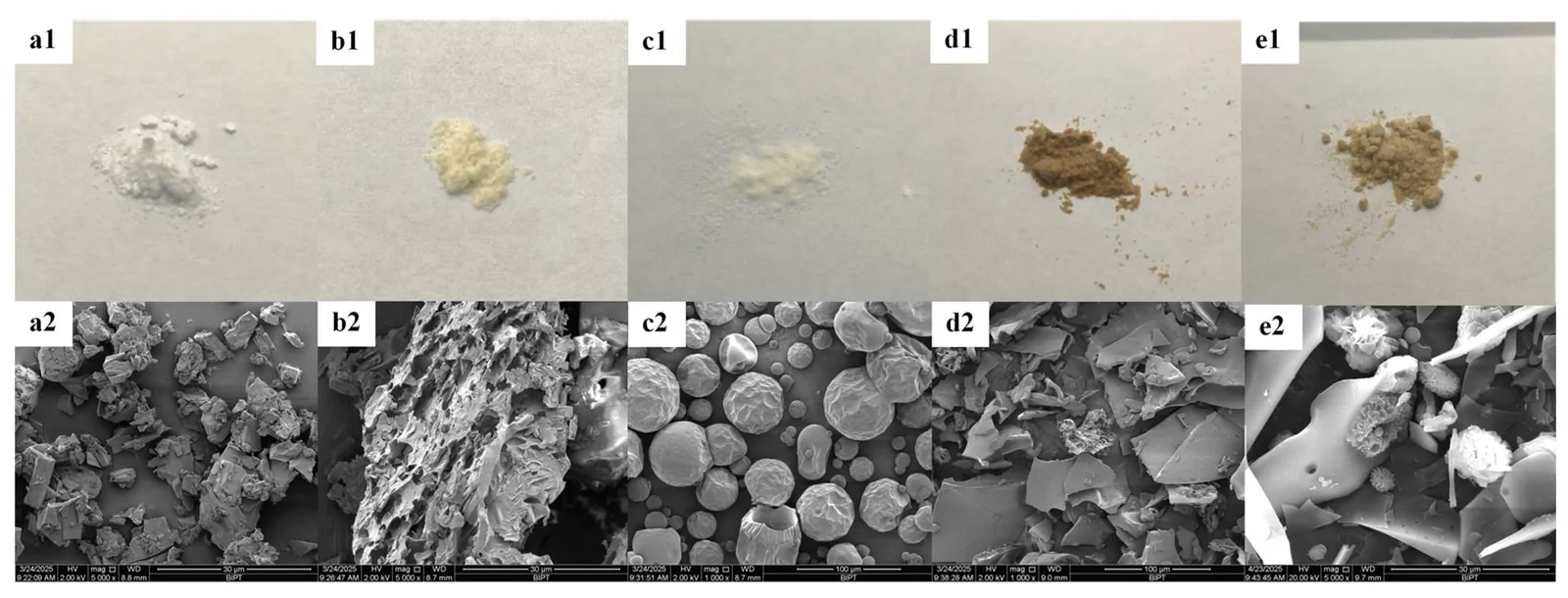
The powder state of the sample and the microstructure observed by SEM. The samples photos are (**a1**) phillyrin standard, (**b1**) forsythoside A standard, (**c1**) chitosan powder, (**d1**) FLE-W, (**e1**) FSE-C and the SEM images of samples are (**a2**) phillyrin standard, (**b2**) forsythoside A standard, (**c2**) chitosan powder, (**d2**) FLE-W, (**e2**) FSE-C.

The macroscopic morphologies of the samples are presented in Figure 8(a1–e1). Phillyrin was observed as a white crystalline powder, whereas forsythoside A exhibited a pale-yellow color. Chitosan presented as an off-white powder. Both the water-extracted *Forsythia suspensa* leaves (FLE-W) and the chitosan-assisted extract (FLE-C) appeared as brown powders, with the latter displaying a noticeably lighter shade.

The microstructures of the respective samples are depicted in Figure 8(a2–e2). As revealed by SEM imaging, phillyrin consists of fine irregular particles, whereas forsythoside A exhibits irregular and porous particles. In contrast, chitosan is characterized by a spherical morphology. The water extract of *Forsythia suspensa* leaves (FLE-W) displayed a coexistence of particulate structures corresponding to both phillyrin and forsythoside A. Notably, in the chitosan-assisted extract (FLE-C), the original particulate structures of the active constituents were no longer preserved. Instead, a novel microstructure emerged, suggesting the formation of new complexes between the bioactive compounds from *Forsythia suspensa* leaves and chitosan. This observation thereby provides direct evidence of an interaction between them. Collectively, the SEM results offer robust support for the analyses derived from the aforementioned FT-IR spectra, PXRD patterns, and other characterizations.

### 2.6. Binding Analysis of Complexes Through Molecular Docking

**Figure 9:**
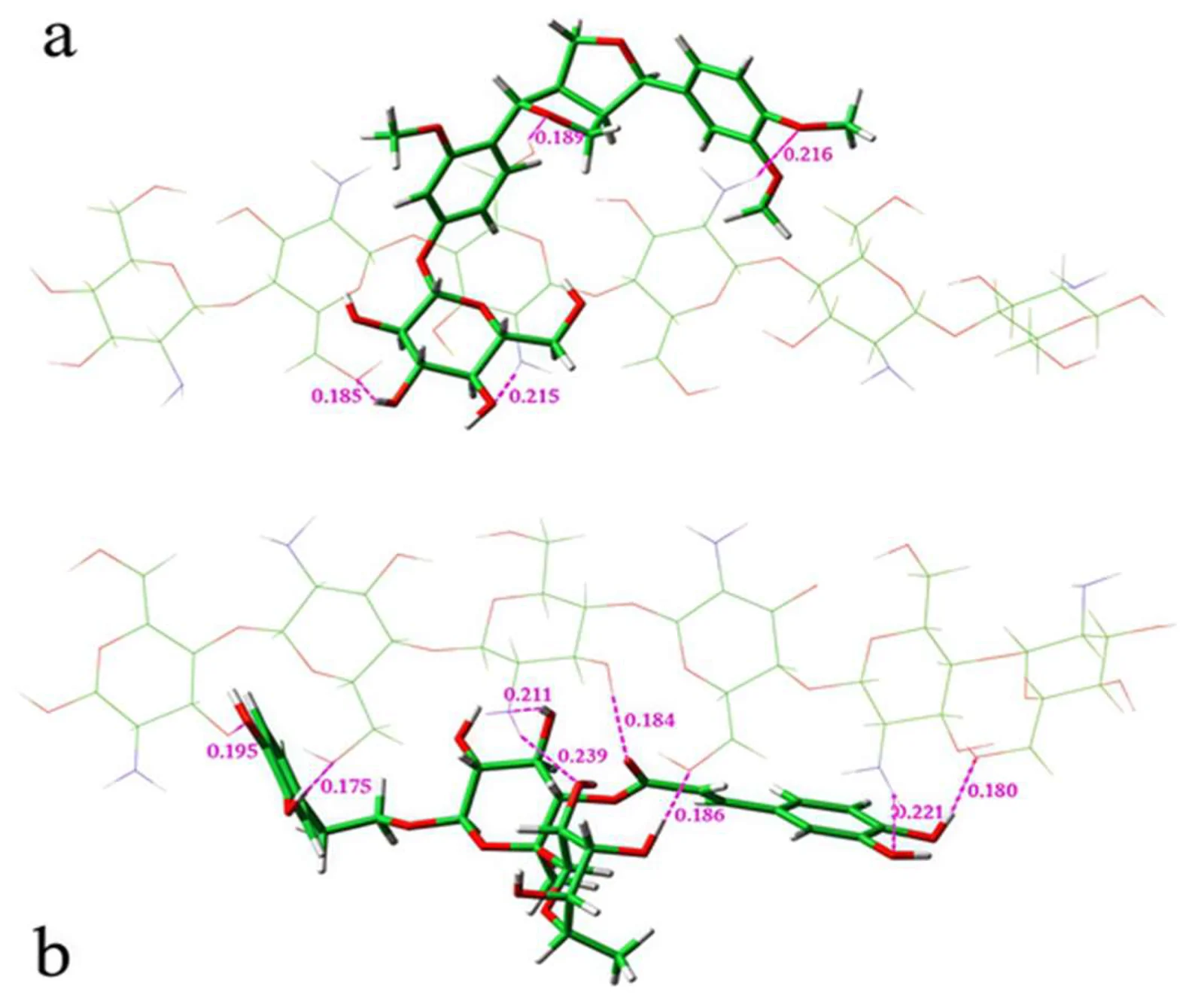
Molecular docking results: (**a**) phillyrin–chitosan (*n* = 4); (**b**) forsythoside A–chitosan (*n* = 4).

To elucidate the molecular interactions between phillyrin, forsythoside A, and chitosan within the inclusion complexes, a molecular docking analysis was conducted. The results confirmed that both phillyrin and forsythoside A are capable of forming inclusion complexes with chitosan at a 1:4 stoichiometric ratio, as illustrated in Figure 9. The intermolecular interaction energies for the phillyrin–chitosan (*n* = 4) and forsythoside A–chitosan (*n* = 4) systems were determined to be 118.7 kJ·mol^−1^ and 108.0 kJ·mol^−1^, respectively. Furthermore, phillyrin and forsythoside A formed four and eight intermolecular hydrogen bonds with chitosan, respectively. Hydrogen bonding plays a pivotal role in stabilizing these inclusion complexes. These findings provide a molecular-level explanation for the enhanced extraction yields of phillyrin and forsythoside A observed in the chitosan-assisted extraction process, as well as for the significant improvement in the water solubility of the FLE-C complex.

### 2.7. Thermal Stability Experimental Results

**Figure 10:**
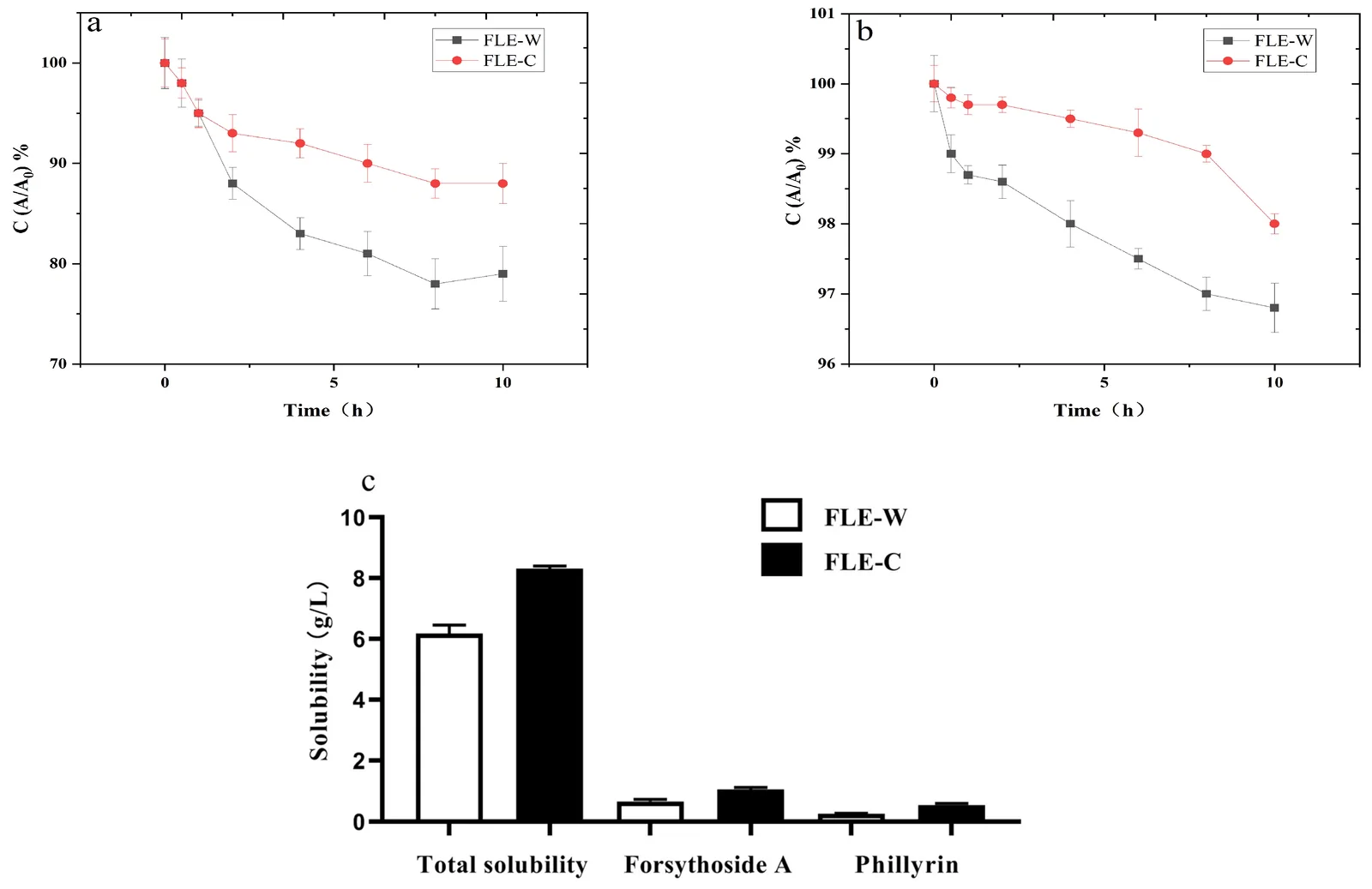
Thermal stability and solubility study of extraction of *Forsythia suspensa* leaves: (**a**) phillyrin; (**b**) forsythoside A; (**c**) water solubility of phillyrin and forsythoside A.

Forsythoside A, owing to its ester bond, is particularly susceptible to thermal degradation in solution. As illustrated in Figure 10a,b, the thermal stability of the bioactive ingredients from *Forsythia suspensa* leaves was investigated. In the aqueous extract, forsythoside A exhibited a sharp decline in concentration, whereas phillyrin degraded at a comparatively slower rate. In contrast, both forsythoside A and phillyrin demonstrated significantly slower degradation kinetics in the chitosan-assisted extract solution compared to the aqueous system. This enhanced stability is likely attributable to molecular interactions between the bioactive ingredients and chitosan, which serve to stabilize their chemical structures.

### 2.8. Water Solubility Experimental Results

The solubility of the *Forsythia suspensa* leaves extracts obtained through aqueous (FLE-W) and chitosan-assisted (FLE-C) extraction is presented in Figure 10c. Upon reaching supersaturation in an aqueous solution at room temperature, the solubility of FLE-C was determined to be 8.32 ± 0.27 g/L, significantly higher than that of FLE-W, which was 6.19 ± 0.35 g/L. This enhancement was further corroborated at the individual compound level. In the FLE-C solution, the concentrations of forsythoside A and phillyrin reached 1.06 ± 0.09 g/L and 0.56 ± 0.06 g/L, respectively. These values represent substantial increases of 58.21% and 115.38% compared to their corresponding solubilities of 0.67 ± 0.06 g/L and 0.26 ± 0.03 g/L in the FLE-W solution. Collectively, these pronounced improvements in solubility provide direct evidence that chitosan interacts with the active ingredients of *Forsythia suspensa* leaves during the extraction process, leading to the formation of novel complexes that enhance the aqueous solubility of these bioactive compounds.

## 3. Materials and Methods

### 3.1. Materials and Reagents

Dried leaves of *Forsythia suspensa* (Thunb.) were supplied by Linyi Jichi Agricultural Science and Technology Co., Ltd. (Linyi, China). The plant material was ground into powder with an electric crusher (SL-100, Yongkang Songqing Hardware Factory, Yongkang, China) and passed through a 40-mesh sieve prior to use. Chitosan was purchased from Jina Sanhe Biotechnology Co., Ltd. (Jinan, China), which had a degree of deacetylation of 0.75 and average molecular weights of 560 KD. For pH modulation throughout the extraction procedure, pharmaceutical-grade glycyrrhizic acid (≥98% purity, Aladdin Reagent Co., Shanghai, China) was employed as the buffering agent. The reference standards, phillyrin, and forsythoside A were obtained from Chengdu Efa Biotechnology Co., Ltd. (Chengdu, China). All other chemical reagents used were of analytical grade and purchased from local suppliers.

### 3.2. Quantification of Phillyrin and Forsythoside A Using High Performance Liquid Chromatography (HPLC)

The concentrations of phillyrin and forsythoside A were quantified using a Waters Alliance HPLC system (Model E2695, Waters Corporation, Milford, MA, USA). Chromatographic separation was achieved on a Waters C18 column (150 mm × 4.6 mm, 5 μm particle size) at a constant temperature of 25 °C. The mobile phase consisted of methanol (solvent A) and water (solvent B) at a flow rate of 1.0 mL/min. The injection volume was 20 μL, and the detection wavelength was set at 235 nm. A detailed gradient elution program is presented in Table 8. Prior to injection, all samples were filtered through a 0.45 μm membrane filter.

### 3.3. Optimization of Extraction Using RSM and NSGA II

The extraction of bioactive ingredients from *Forsythia suspensa* leaves was performed as follows: First, 10 g of dried *Forsythia suspensa* powder, pulverized and passed through a 40-mesh sieve, was mixed with a chitosan solution. The mixture was then subjected to heat-reflux extraction under optimized conditions. Immediately upon completion, the crude extract was separated from the solid residue via vacuum filtration. The resulting filtrate was cooled to ambient temperature and filtered again through a 0.45 μm membrane. An aliquot of the final filtrate was reserved for HPLC analysis, while the remainder was freeze-dried to obtain a solid powder for the subsequent experiments.

The key extraction parameters, which are the extraction time, solid-to-liquid ratio (R_S/L_), extraction temperature, chitosan dosage, and pH, were systematically optimized using a combination of single-factor experiments and response surface methodology (RSM). In the single-factor experimental design, each parameter was investigated at five distinct levels: extraction time (60, 75, 90, 105, and 120 min), solid-to-liquid ratio (R_S/L_) (1:10, 1:30, 1:50, 1:70, and 1:90 g/mL), extraction temperature (50, 60, 70, 80, and 90 °C), chitosan dosage (2.5, 5.0, 7.5, 10.0, and 12.5 g/10 g *Forsythia suspensa* leaves), and pH (3.5, 4.0, 4.5, 5.0, and 5.5).

Following the identification of the key process variables via one-factor-at-a-time (OFAT) experiments, a four-factor response surface methodology (RSM) was employed to model the relationship between the response variables and these process parameters. Based on the preliminary optimization results, a Box–Behnken Design (BBD) was implemented to systematically investigate the effects of four independent variables: extraction time (X_1_), ratio of solid to liquid (R_S/L_, X_2_), extraction temperature (X_3_), and chitosan dosage (X_4_). The values and levels of the individual variables are provided in Table 9, with −1 rep-resenting the low level, 1 the high level, and 0 the central level. The extraction efficiency equation is shown below:

(3) $$Extraction\;yield\;of\;Phillyrin\left(\%\right)=\frac{mass\;of\;extracted\;Phillyrin}{mass\;of\;added\;Forsythia\;leaves}\times 100\%;$$

(4) $$Extraction\;yield\;of\;forsythoside\;A\left(\%\right)=\frac{mass\;of\;extracted\;Forsythoside\;A}{mass\;of\;added\;Forsythia\;leaves}\times 100\%.$$

The response surface experiment values and levels are shown in Table 4. After the Box–Behnken design, a total of 29 sets of experiments including 5 sets of median conditions were completed.

To identify the optimal ranges for the extracted parameters and the corresponding optimization objectives, the Non-dominated Sorting Genetic Algorithm II (NSGA-II) was employed. This facilitated the development of a multi-objective optimization model, mathematically formulated in Equation (5), with constraints derived from the experimental parameter ranges previously established, as detailed in Equation (6).

(5) $$\emptyset_{\left(1,2\right)}=\left\{\begin{array}{c} \emptyset_{1}=max\left[Y_{1}(X_{1}{,X}_{2}{,X}_{3}{,X}_{4})\right]\\ \emptyset_{2}=max\left[Y_{2}(X_{1}{,X}_{2}{,X}_{3}{,X}_{4})\right] \end{array}\right.$$

(6) $$s.t.\left\{\begin{array}{c} {60\leq X}_{1}\leq 120\;min\\ 1:30\leq X_{2}\leq 1:70\\ 70\leq X_{3}\leq 90\;{}^{\circ}C\\ 2.5\leq X_{4}\leq 12.5\;g \end{array}\right.$$

The optimal extraction parameters were determined using the NSGA-II algorithm. To validate these conditions, the extraction procedure was performed in triplicate. For comparison, a control experiment was also conducted in triplicate using pure water under otherwise identical conditions. The results from these two sets of experiments were then compared to evaluate the enhancing effect of chitosan.

### 3.4. Characterization of Chitosan-Assisted Extraction of Natural Products from Forsythia suspensa Leaves

#### 3.4.1. Fourier Transform Infrared Spectroscopy (FT-IR)

For Fourier Transform Infrared (FT-IR) Spectroscopy, each sample—including the water extract of *Forsythia suspensa* leaves (FLE-W), the chitosan-assisted extract (FLE-C), phillyrin standard, forsythoside A standard, and chitosan—was homogenized with spectroscopic-grade potassium bromide (KBr) and subsequently pressed into a thin pellet. FT-IR spectra were recorded on a TENSOR 27 spectrometer (Bruker, Karlsruhe, Germany) in the range of 4000–500 cm^−1^, with a spectral resolution of 4 cm^−1^. Each spectrum represents an average of 16 successive scans to enhance the signal-to-noise ratio.

#### 3.4.2. Powder X-Ray Diffraction (PXRD)

X-ray diffraction (XRD) patterns were recorded to analyze the crystalline structure of the samples, including the water extract of *Forsythia suspensa* leaves (FLE-W), the chitosan-assisted extract (FLE-C), phillyrin standard, forsythoside A standard, and chitosan. The analysis was performed on an X-ray diffractometer (Rigaku Corporation, Tokyo, Japan) equipped with a Cu Kα radiation source, operating at 40 kV and 120 mA. Data were collected over a 2θ range of 5° to 80° with a step size of 0.02°.

#### 3.4.3. Differential Scanning Calorimetry (DSC)

The DSC analysis of samples was performed using a SDT650 simultaneous DSC-TGA thermal analyzer (TA Instruments, New Castle, DE, USA). For each measurement, approximately 5 mg of the sample, including the chitosan-assisted extract (FLE-C), phillyrin standard, forsythoside A standard, and chitosan, was placed in a sealed alumina crucible and heated from ambient temperature to 500 °C at a heating rate of 10 °C/min under a continuous nitrogen purge (flow rate: 50 mL/min). Before conducting measurements, the differential scanning calorimeter (DSC) was calibrated with benzoic acid (certified reference material, purity > 99.9%) following the standardized protocol outlined in ASTM E967-18 [38].

#### 3.4.4. Scanning Electron Microscopy (SEM)

The surface morphology of the samples, including the water extract of *Forsythia suspensa* leaves (FLE-W), the chitosan-assisted extract (FLE-C), phillyrin standard, forsythoside A standard, and chitosan, were investigated using a Quanta 400 field emission scanning electron microscope (FEI, Hong Kong, China). For sample preparation, each specimen was dispersed onto an aluminum stub coated with conductive carbon tape. Excess loosely adhered particles were gently removed using a stream of compressed air. Subsequently, the samples were sputter-coated with a thin layer of gold (~10 nm) to enhance conductivity prior to imaging.

### 3.5. Molecular Docking

To elucidate the interaction mechanism between the components, quantum chemical calculations were performed using the ORCA 5.0 program package [36] and the Multiwfn code [39]. A chitosan oligomer with a degree of polymerization (DP) of 4 was employed as the model system. All geometry optimizations and energy calculations were carried out using the hybrid density functional theory (DFT) method B3LYP, which was augmented with Grimme’s D3 dispersion correction [40,41] in conjunction with the def2-TZVP basis set. The interaction energy between the constituent moieties was determined using the supramolecular approach, wherein the binding energy (ΔE) is calculated as the difference between the energy of the complex and the sum of the energies of the isolated monomers: ΔE = E^AB^ − (E^A^ + E^B^) [42].

### 3.6. Water Solubility Experiment

The equilibrium solubility of the water extract of *Forsythia suspensa* leaves (FLE-W) and the chitosan-assisted extract (FLE-C) was determined using the shake-flask method. In brief, an excess amount of each powder was separately added to 100 mL of deionized water in individual vessels. The resulting suspensions were stirred at room temperature for 60 min to reach dissolution equilibrium. Subsequently, the suspensions were centrifuged at 4000 rpm for 5 min. The supernatant from each sample was carefully collected and filtered through a 0.22 μm membrane filter to remove any undissolved particles. To ensure reliability, the entire experiment was performed in triplicate for each sample. The concentrations of phillyrin and forsythoside A in the final saturated solutions were then quantified using high performance liquid chromatography (HPLC).

### 3.7. Thermal Stability Experiment

To evaluate the thermal stability of the target compounds, aliquots (100 mL) of the saturated aqueous solution, prepared as detailed in Section 3.6, were incubated in a water bath maintained at 90 °C for varying durations (0, 0.5, 1, 2, 4, 6, 8, and 10 h). Following the heat treatment, the samples were allowed to cool to room temperature. Subsequently, the concentrations of phillyrin and forsythoside A in each sample were quantified using high performance liquid chromatography (HPLC).

### 3.8. Statistical Analysis

The experimental design for the response surface methodology (RSM) and the subsequent analysis of the data were performed using Design-Expert software (version 8.0.6, Stat-Ease, Inc., Minneapolis, MN, USA). All other statistical analyses and graphical representations were conducted using Origin 2021 (OriginLab Corporation, Northampton, MA, USA) and GraphPad Prism 8 (GraphPad Software, San Diego, CA, USA).

## 4. Conclusions

This study presents a novel chitosan-assisted extraction method for phillyrin and forsythoside A, the primary bioactive constituents from *Forsythia suspensa* leaves. This methodology capitalizes on the intrinsic mucoadhesive characteristics of chitosan, facilitating selective molecular interactions with target bioactive ingredients to form a stabilized extractant complex. The formation of this inclusion complex was conclusively verified through a suite of analytical techniques, including Powder X-ray Diffraction (PXRD), Fourier Transform Infrared Spectroscopy (FT-IR), Differential Scanning Calorimetry (DSC), Scanning Electron Microscopy (SEM), and molecular docking simulations. Furthermore, the experimental data demonstrated that the presence of chitosan significantly enhanced the stability and aqueous solubility of the extracted bioactive compounds. To systematically optimize the extraction parameters, three distinct strategies were employed: orthogonal design, response surface methodology (RSM), and the Non-dominated Sorting Genetic Algorithm II (NSGA-II). RSM was effectively utilized to determine the optimal conditions for maximizing the yield of each individual compound (phillyrin or forsythoside A). In contrast, NSGA-II was applied as a multi-objective optimization tool to identify the Pareto-optimal set of conditions that simultaneously maximize the yields of both phillyrin and forsythoside A. This comparative study provides an effective and insightful exploration into the establishment of optimal conditions for the concurrent extraction of multiple bioactive ingredients from natural products. This innovative chitosan-assisted extraction technology holds great promise as a valuable tool for the natural product industry and is poised to accelerate research in the food, pharmaceutical, cosmetic, and nutraceutical sectors, as well as in the investigation of other valuable botanical species.

In this study, chitosan was strategically selected as an extraction adjuvant owing to its exceptional biocompatibility and multifunctionality, which are markedly superior to conventional bi-polymers (e.g., alginate, cellulose derivatives). The inherent cationic polyelectrolyte characteristics of chitosan enable selective electrostatic interactions with anionic bioactive compounds, while its tunable degree of deacetylation provides precise modulation of solubility parameters and intermolecular binding affinities. Notably, chitosan exhibits dual functionality as both a molecular adsorbent and a natural coagulant, thereby significantly reducing the reliance on synthetic processing aids and fully complying with contemporary sustainable extraction principles [43]. In this study, the extraction yields of phillyrin and forsythoside A obtained by the chitosan-assisted extraction method were determined to be 1.68 ± 0.16% and 3.23 ± 0.27%, respectively. Notably, these values represent a significant improvement compared to those obtained by conventional water extraction. Furthermore, the extraction efficiencies achieved with this method were comparable to those reported for ethanol extraction [35] and microwave-assisted extraction techniques [13].

Although this study demonstrated that chitosan-assisted extraction significantly enhances the yields of phillyrin and forsythoside A, subsequent purification steps (such as pH adjustment, resin adsorption, or gel chromatography) required for isolating these bioactive ingredients from chitosan may introduce additional process complexity and potentially reduce the overall yield. Furthermore, beyond phillyrin and forsythoside A, *Forsythia suspensa* leaves contain abundant polyphenolic compounds such as rutin and chlorogenic acid. While our findings confirmed the selective extraction of target compounds (phillyrin and forsythoside A) by chitosan, the synergistic extraction mechanisms for other polyphenols warrant systematic investigation. Additionally, comprehensive evaluation is needed to determine whether the bioactivities (e.g., antioxidant capacity as assessed by DPHH radical scavenging assay) of the target compounds remain comparable to their conventionally extracted counterparts after dissociation from chitosan complexes. Future work will focus on method validation, robustness testing, and assessing the technique’s capacity for simultaneous extraction and isolation of multiple bioactive constituents from natural products, thereby advancing the development of integrated extraction technologies.

## Figures and Tables

**Table 1:** Box–Behnken design with the independent variables.

	X_1_	X_2_	X_3_	X_4_	Phillyrin (Y_1_, Yield, %)	Forsythoside A (Y_2_, Yield, %)
1	0	0	0	0	1.06	2.58
2	1	0	−1	0	0.42	0.19
3	0	1	1	0	1.56	2.28
4	−1	−1	0	0	0.81	2.17
5	1	0	0	−1	1.02	1.31
6	0	−1	−1	0	0.29	0.16
7	0	1	−1	0	0.62	0.50
8	0	1	0	0	1.20	1.54
9	1	−1	0	0	0.93	1.73
10	1	0	1	0	1.58	3.03
11	0	0	0	0	1.06	2.64
12	0	0	0	0	1.09	2.60
13	1	0	0	0	1.19	2.22
14	0	0	−1	−1	0.57	0.56
15	0	−1	1	0	1.32	2.79
16	0	−1	0	0	0.65	1.76
17	0	0	1	0	1.45	3.25
18	0	1	0	−1	1.06	1.88
19	−1	0	1	0	1.58	3.08
20	−1	0	−1	0	0.47	0.24
21	0	−1	0	−1	0.78	2.02
22	0	0	0	0	1.06	2.62
23	0	0	−1	0	0.42	0.19
24	0	0	0	0	1.05	2.61
25	−1	0	0	−1	1.10	2.52
26	−1	0	0	0	1.09	1.29
27	1	1	0	0	1.39	2.48
28	−1	1	0	0	1.13	1.37
29	0	0	1	−1	1.38	3.16

**Table 2:** Analysis of variance (ANOVA) for the fitted quadratic polynomial model of phillyrin.

Variance Source	Sum of Squares	Mean Square	F-Value	*p*-Value Rob > F	Significance
Model	3.64	0.26	43.92	<0.0001	Yes
Residual	0.083	5.92 × 10^−3^	—	—	—
R-squared	0.9777	Adeq precision	24.958		

**Table 3:** Analysis of variance (ANOVA) for the fitted quadratic polynomial model of forsythoside A.

Variance Source	Sum of Squares	Mean Square	F-Value	*p*-Value Rob > F	Significance
Model	26.35	1.88	41.27	<0.0001	Yes
Residual	0.64	0.046	—	—	—
R-squared	0.9763	Adeq precision	19.878		

**Table 4:** Regression coefficients of the quadratic polynomial model of phillyrin.

Parameter	Sum of Squares	Mean Square	F-Value	*p*-Value	Significance
X_1_	0.011	1	0.011	1.85	--
X_2_	0.39	0.39	66.17	<0.0001	***
X_3_	3.09	3.09	521.21	<0.0001	***
X_4_	7.40 × 10^−4^	7.40 × 10^−4^	0.13	0.7289	--
X_1_X_2_	4.61 × 10^−3^	4.61 × 10^−3^	0.78	0.3923	--
X_1_X_3_	8.37 × 10^−4^	8.37 × 10^−4^	0.14	0.7125	--
X_1_X_4_	9.06 × 10^−3^	9.06 × 10^−3^	1.53	0.2364	--
X_2_X_3_	1.67 × 10^−3^	1.67 × 10^−3^	0.28	0.6034	--
X_2_X_4_	0.018	0.018	3.12	0.0993	--
X_3_X_4_	0.012	0.012	2.07	0.1723	--
X_1_^2^	0.021	0.021	3.5	0.0822	--
X_2_^2^	0.027	0.027	4.57	0.0507	--
X_3_^2^	0.037	0.037	6.23	0.0257	**
X_4_^2^	0.012	0.012	2.09	0.1703	*
Model	3.64	0.26	43.92	<0.0001	***

* significant at 0.05 level; ** significant at 0.01 level; *** significant at 0.001 level.

**Table 5:** Regression coefficients of the quadratic polynomial model of forsythoside A.

Parameter	Sum of Squares	Mean Square	F-Value	*p*-Value	Significance
X_1_	6.89 × 10^−3^	6.89 × 10^−3^	0.15	0.7033	--
X_2_	0.028	0.028	0.62	0.4455	--
X_3_	20.65	20.65	452.89	<0.0001	***
X_4_	0.12	0.12	2.65	0.1259	--
X_1_X_2_	0.6	0.6	13.09	0.0028	**
X_1_X_3_	3.19 × 10^−6^	3.19 × 10^−6^	7.00 × 10^−5^	0.9934	--
X_1_X_4_	1.14	1.14	25.06	0.0002	***
X_2_X_3_	0.18	0.18	4.04	0.0641	--
X_2_X_4_	1.41 × 10^−3^	1.41 × 10^−3^	0.031	0.8631	--
X_3_X_4_	0.054	0.054	1.18	0.2961	--
X_1_^2^	0.75	0.75	16.44	0.0012	**
X_2_^2^	1.36	1.36	29.83	<0.0001	***
X_3_^2^	2.45	2.45	53.65	<0.0001	***
X_4_^2^	0.71	0.71	15.5	0.0015	**
Model	26.35	1.88	41.27	<0.0001	***

** significant at 0.01 level; *** significant at 0.001 level.

**Table 6:** Optimization results from response surface methodology and NSGA-II.

Optimization Method	Optimized Extraction Parameters				Predicted Yield	
	X_1_	X_2_	X_3_	X_4_	Phillyrin	Forsythoside A
RSM Equation (1)	120.00	1:67.46	80.50	9.52	1.84%	
RSM Equation (2)	88.25	1:51.23	81.00	8.34		3.34%
NSGA-II	119.99	1:52.06	79.99	11.75	1.69%	3.23%

**Table 7:** Experimental results of the extraction with and without chitosan (*n* = 3).

Method	Extraction Parameters				Yield	
	X_1_	X_2_	X_3_	X_4_	Phillyrin	Forsythoside A
Without chitosan	120	1:52	80	0	0.32 ± 0.12%	1.55 ± 0.22%
NSGA-II optimization of chitosan-assisted	120	1:52	80	11.75	1.68 ± 0.16%	3.23 ± 0.27%

**Table 8:** Phillyrin and forsythoside A gradient elution conditions.

Time (min)	A (%)	B (%)	Gradient Curve
0–10	10→25	90→75	6
10–40	25→40	75→60	6
40–60	40→60	60→40	6

**Table 9:** Experimental values and levels.

Level	Test Factors			
	Extraction Time (min)	The Ratio of Solid to Liquid (g/mL)	Extraction Temperature (°C)	Chitosan Dosage (g/g)
−1	60	1:30	70	10:2.5
0	90	1:50	80	10:7.5
1	120	1:70	90	10:12.5

## Data Availability

The original contributions presented in this study are included in the article. Further inquiries can be directed to the corresponding author.
